# Comparison of radioactive and non-radioactive iothalamate and hippuran to assess kidney function

**DOI:** 10.1093/ckj/sfaf293

**Published:** 2025-09-23

**Authors:** Abdulfataah A A Mohamed, Ron T Gansevoort, Nico C van de Merbel, Marco van Londen, Martin H de Borst, Rolf Zijlma, Lenneke A T Junier, Hiddo J L Heerspink, Jasper Stevens

**Affiliations:** Department of Clinical Pharmacy and Pharmacology, University of Groningen, University Medical Center Groningen, Groningen, The Netherlands; Department of Nephrology, University of Groningen, University Medical Center Groningen, Groningen, The Netherlands; Department of Nephrology, University of Groningen, University Medical Center Groningen, Groningen, The Netherlands; ICON Bioanalytical Laboratories, Assen, The Netherlands; Department of Analytical Biochemistry, University of Groningen, Groningen, The Netherlands; Department of Nephrology, University of Groningen, University Medical Center Groningen, Groningen, The Netherlands; Department of Nephrology, University of Groningen, University Medical Center Groningen, Groningen, The Netherlands; Department of Nuclear Medicine and Molecular Imaging, University of Groningen, University Medical Center Groningen, Groningen, The Netherlands; Department of Clinical Pharmacy and Pharmacology, University of Groningen, University Medical Center Groningen, Groningen, The Netherlands; Department of Clinical Pharmacy and Pharmacology, University of Groningen, University Medical Center Groningen, Groningen, The Netherlands; Department of Clinical Pharmacy and Pharmacology, University of Groningen, University Medical Center Groningen, Groningen, The Netherlands

**Keywords:** ERPF, hippuran, iothalamate, kidney function, mGFR

## Abstract

**Background:**

Kidney function can be assessed by the measured glomerular filtration rate (mGFR) and effective renal plasma flow (ERPF) using the exogenous filtration markers ^125^I-iothalamate and ^131^I-hippuran. These markers are unfavourable due to the radioactive burden for patients, personnel and the environment. We studied whether we could replace the measurement of these radiolabelled compounds (‘warm method’) with the measurement of their non-radioactive isotopologues (‘cold method’).

**Methods:**

We determined mGFR and ERPF in 220 participants by both the warm (gamma counting) and cold (liquid chromatography–tandem mass spectrometry) methods on the same serum, urine and infusion solution samples. Agreement between the methods was evaluated using Passing–Bablok regression and Bland–Altman analysis. Accuracy criteria were that ≥80% of the warm and cold mGFR measurements were within ±30% (P_30_) and ≥50% within ±10% (P_10_). Precision of mGFR was assessed by the standard deviation (SD) of the bias and the intratest coefficient of variation (CV%) of the measurement methods were determined.

**Results:**

mGFR measurements showed a mean difference of 1.97% and no clinically relevant bias when comparing the warm and cold methods. mGFR was accurate with a P_30_ of 100% and a P_10_ of 76%. The cold method was precise; the SD was 8.04 ml/min and the intratest CV% was 3.01 ± 3.15%. The ERPF values showed a mean difference of 19.2% and a large constant and proportional bias. Radiochemical impurities, which influence the warm method, were found in the ^131^I-hippuran formulation, and these were the cause of the discrepancy between the two methods.

**Conclusions:**

The cold method provides equivalent mGFR results compared with the warm method. The ERPF determined using the cold method is unaffected by radiochemical impurities that significantly affect the warm method.

## GRAPHICAL ABSTRACT

**GRAPHICAL ABSTRACT:**
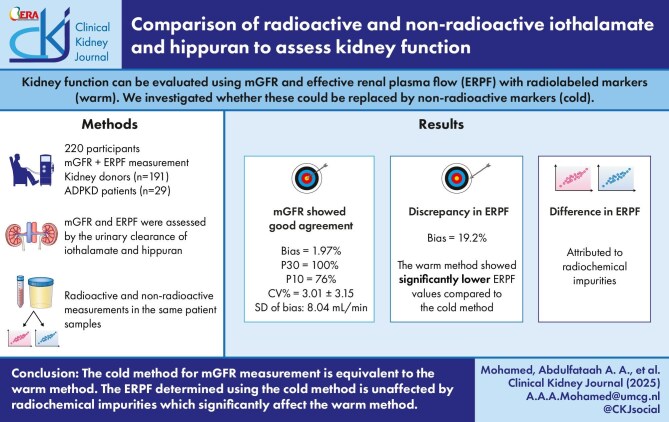

KEY LEARNING POINTS **What was known:**

- Measured glomerular filtration rate (mGFR) and effective renal plasma flow (ERPF) can be measured using radiolabelled iothalamate and hippuran, which has the disadvantage of relying on radioactive compounds.

**This study adds:**

- This study demonstrates that by measuring non-radioactive iothalamate and hippuran it is possible to accurately determine mGFR.
- Discrepancies in ERPF between radioactive and non-radioactive hippuran measurements are due to impurities in the radioactive hippuran formulation.
- The cold method is not susceptible to interference from impurities.

**Potential impact:**

- These results demonstrate that the non-radioactive measurement method is a viable option to determine the mGFR.
- Non-radioactive measurement of kidney function reduces the reliance on radioactive substances, thereby lowering the burden on patients, personnel and the environment, which encourages broader implementation of exact renal function assessment.

## INTRODUCTION

In clinical practice, the glomerular filtration rate (GFR) is estimated using creatinine and equations to assess kidney function. The Kidney Disease: Improving Global Outcomes guidelines recommend the use of exogenous filtration markers to determine GFR in situations where assessment with endogenous filtration markers is not sufficiently accurate [1]. These circumstances include, for instance, confirming the diagnosis of chronic kidney disease in patients where creatinine-based estimated GFR (eGFR) may be unreliable, determining eligibility for kidney donation and dose adjustments for renally excreted drugs with a narrow therapeutic index [1–3]. In addition, the European Medicines Agency recommends measured GFR (mGFR) during the drug development process to accurately assess the effect of drugs on kidney function [4, 5].

Determining the urinary clearance of inulin is considered the gold standard for mGFR assessment since inulin is freely filtered, not bound to protein, not reabsorbed nor secreted by the tubule and not metabolized by the kidney or any other organ and thus the urinary clearance of inulin is equal to the GFR [6]. Inulin, however, is no longer available for use in humans. Extensive evidence supports that assessing the clearance of iothalamate also provides an accurate mGFR, closely aligning with values obtained by assessing inulin clearance [6–8]. Notably, urinary clearance methods offer superior accuracy over plasma clearance approaches, particularly in cases where plasma-based methods are biased. These include situations involving extrarenal elimination of the filtration marker, which may lead to overestimation of GFR; glomerular hyperfiltration, where early compartment correction may result in underestimation; and large changes in extracellular fluid volume, such as volume expansion or contraction, which can cause either overestimation or underestimation of GFR [8–12]. Measurement of GFR by assessing the urinary clearance of ^125^I-iothalamate and ^131^I-hippuran has a high accuracy and precision when compared with measurement of GFR by assessing inulin [6–8]. It has therefore been used in the clinic for decades [13, 14]. This technique is further referred to as the ‘warm method’, because it involves the use of radiolabelled compounds and quantification of radioactivity in blood and urine samples using a gamma counter. Co-administration of hippuran allows for the simultaneous measurement of effective renal plasma flow (ERPF), which can be determined in serum and in urine. The ratio of these is used to correct for inaccuracies in urine collection, thereby improving the accuracy of the mGFR as described by Apperloo *et al.* [14].

Despite its high accuracy and precision, the warm method has several disadvantages, such as the radioactive burden for patients, personnel and the environment, the limited shelf life caused by radioactive decay and the high cost of materials. Ideally, a non-radioactive version of this mGFR method (a ‘cold method’) is needed to maintain the ability to measure GFR accurately [15].

Previously we developed sensitive liquid chromatography–tandem mass spectrometry (LC-MS/MS) methods for the measurement of trace amounts of non-radioactive iothalamate and hippuran in serum and urine that potentially meets this need [16]. In this study we compare the warm and cold methods in the same patient samples. We hypothesized that the cold method would show sufficiently high accuracy and precision in the measurement of GFR and ERPF compared with the warm method and that it could therefore serve as a valid non-radioactive alternative.

## MATERIALS AND METHODS

### Study design and participants

The 220 participants in this study were living kidney donors (*n* = 191) and patients with autosomal dominant polycystic kidney disease (ADPKD; *n* = 29) who all received radiolabelled ^125^I-iothalamate and ^131^I-hippuran to determine mGFR between 17 July 2021 and 10 February 2022 in the framework of studies approved by the Institutional Review Board of the University Medical Center Groningen (METc 2014/077 and METc 2013/040) [17]. These studies followed the ethical standards of the World Medical Association Declaration of Helsinki and were conducted in adherence with the International Conference on Harmonization Good Clinical Practice guidelines. The medical records of these patients were reviewed for demographic information and lab results.

### Procedure

GFR and ERPF were measured using the simultaneous infusion of radiolabelled ^125^I-iothalamate (t_1/2_: 59 days) and ^131^I-hippuran (t_1/2_: 8 days), as previously described [13, 18]. In short, a priming dose was administered intravenously containing 0.3 MBq of ^125^I-iothalamate (Glofil-125, Iso-Tex Diagnostics, Pearland, TX, USA) and 0.4 MBq of ^131^I-hippuran (Hippuran, Polatom, Poland). Immediately thereafter, a continuous infusion of both tracers was started at concentrations of 0.0075 MBq/ml ^125^I-iothalamate and 0.02 MBq/ml ^131^I-hippuran. Per patient, the individual infusion rate was adjusted based on serum creatinine levels to ensure an appropriate steady-state concentration of the tracers: 6, 9 or 12 ml/h for serum creatinine levels >200, 100–200 or <100 µmol/L, respectively.

After a stabilization period of 90 minutes, blood samples were drawn at 90, 150, 210, 270 and 330 minutes. Blood was collected in serum tubes (Vacuette^®^ CAT) and separation was performed immediately after the sampling was completed. Patients were instructed to collect urine in two 2-h periods: at 90–210 minutes and 210–330 minutes after start of the continuous intravenous administration of the radiolabelled tracers.

### Measurement and calculation of GFR and ERPF

Directly after sampling, radioactivity levels of ^125^I-iothalamate and ^131^I-hippuran, expressed in counts per minute (CPM) were measured in serum, urine and infusion solution samples using a 2470 WIZARD^2^ gamma counter (Perkin Elmer, Waltham, MA, USA). Afterwards, all samples were locally stored at −20°C.

The uncorrected mGFR (hereafter simply referred to as mGFR) was determined by calculating the average of the two 2-hour urinary clearance periods of ^125^I-iothalamate:


(1) \begin{equation*}{\mathrm{mGFR}} = \left( {U \times {V}_U} \right)/P,\end{equation*}

where *U* is the activity of ^125^I-iothalamate in urine (CPM), *V*_U_ the urine flow rate (ml/min) and *P* is the activity of ^125^I-iothalamate in serum (CPM).

The ERPF was determined by calculating the urinary clearance of ^131^I-hippuran (ERPF_U_):


(2) \begin{equation*}{\mathrm{ERP}}{{\mathrm{F}}}_{\mathrm{U}} = \left( {U \times {V}_U} \right)/P,\end{equation*}

where *U* is the activity of ^131^I-hippuran in urine (CPM), *V*_U_ the urine flow rate (ml/min) and *P* is the activity of ^131^I-hippuran serum (CPM).

When a steady state of ^131^I-hippuran is achieved the ERPF can also be determined by assessing the serum clearance of ^131^I-hippuran (ERPF_IF_):


(3) \begin{equation*}{\mathrm{ERP}}{{\mathrm{F}}}_{{\mathrm{IF}}} = \left( {I \times {V}_I} \right)/P,\end{equation*}

where *I* is the activity of ^131^I-hippuran (CPM) in the infusion fluid, *V*_I_ the infusion rate (ml/min) and *P* is the activity of ^131^I-hippuran in serum (CPM).

The simultaneous determination of ERPF using both the ERPF_U_ and ERPF_IF_ clearance methods enables correction of urinary clearance of iothalamate for incomplete urine collection [13]. Under conditions of complete urine collection, the clearance of ^131^I-hippuran by urinary and serum clearance is identical [19]. Because ERPF_U_ equals ERPF_IF_, GFR can be corrected for urine collection errors by multiplying the urinary clearance of ^125^I-iothalamate by the ratio of serum to urinary clearance of ^131^I-hippuran:


(4) \begin{equation*}{\mathrm{mGF}}{{\mathrm{R}}}_{{\mathrm{COR}}} = {\mathrm{mGFR}}\left( {{\mathrm{ERP}}{{\mathrm{F}}}_{{\mathrm{IF}}}/{\mathrm{ERP}}{{\mathrm{F}}}_{\mathrm{U}}} \right).\end{equation*}

When ERPF_IF_ is <100 ml/min, extrarenal clearance of hippuran becomes relevant and only the urinary clearance of ^125^I-iothalamate (Equation 1) and urinary clearance of ^131^I-hippuran (Equation 2) are used to determine the mGFR and ERPF_U_.

In 2024, when radioactivity of the samples was negligible, the stored 1100 serum and 440 urine samples of 220 patients were analysed for iothalamate and hippuran concentrations using the previously validated LC-MS/MS methods [16]. In addition, as the original infusion dose was based on radioactivity (in MBq), the original 440 infusion solution samples were analysed by LC-MS/MS to determine the iothalamate and hippuran concentrations (in ng/ml). The analysis of the samples and assessment of long-term storage stability was performed as described in the supplementary material. In short, serum samples were pretreated by protein precipitation with methanol, followed by supernatant dilution, while urine and infusion solution samples were prepared by simple dilution. Iothalamate and hippuran in the pretreated samples were chromatographically separated using a 6.5-minute gradient on an HSS T3 column (Waters, Milford, MA, USA) and detected via electrospray ionization in positive ion mode, with stable isotope–labelled internal standards using a Model 6500 triple quadrupole mass spectrometer (SCIEX, Concord, ON, Canada). Equations 1–4 were used to calculate mGFR, ERPF_U_, ERPF_IF_ and mGFR_COR_, replacing the radioactivity levels in CPM for iothalamate and hippuran for their respective concentrations in ng/ml.

### Measurement of impurities

The presence of impurities in the ^131^I-hippuran formulation was assessed using high-performance liquid chromatography (HPLC) separation with radioactivity detection and their structural identification by (high-resolution) Orbitrap LC-MS and ^1^H nuclear magnetic resonance (NMR) analysis and the methodologies as described in the supplementary material.

### Statistical analysis

The mGFR and ERPF were calculated for both the warm (mGFR_COR_,_warm_, mGFR_warm_ and ERPF_warm_) and cold methods (mGFR_COR_,_cold_, mGFR_cold_ and ERPF_cold_). The precision of mGFR_COR_ and mGFR was assessed by the SD of the bias (between mGFR_warm_ and mGFR_cold_). The 95% confidence intervals (CIs) of the SD were calculated using the bootstrap method (*n* = 2000) and the Pitman–Morgan test was used to compare the precision of mGFR_COR_ and mGFR. In addition, the intratest variability was assessed and this was calculated by comparing the two consecutive 120-minute urine clearance periods and corresponding serum samples as described previously [14]. The intratest coefficient of variation as expressed in percentage (CV%) was reported as the geometric mean of the CV% ± SD. For the geometric mean the values were log transformed to achieve normality, then the mean of these log transformed values was exponentiated to obtain the geometric mean. Two-sided paired Student’s *t*-tests were used to compare the mGFR_COR_ and mGFR for both the warm and cold methods; *P*-values <.05 were considered statistically significant.

Agreement between the warm and cold methods was evaluated by Bland–Altman plots to determine the mean percentage difference [e.g. (mGFR_cold_ − mGFR_warm_)/mean*100%] and 95% limits of agreement. Passing–Bablok regression was used to determine constant and proportional bias. Statistical analyses were performed using Analyse-it version 5.81 for Microsoft Excel (Analyse-it Software, Leeds, UK) and R version 4.4.3 (R Foundation for Statistical Computing, Vienna, Austria).

The accuracy of mGFR_COR_ and mGFR, when comparing the warm and cold methods, were determined and these were considered acceptable when ≥80% of measurements were within ±30% (P_30_) and ≥50% were within ±10% (P_10_) [8]. To determine whether the accuracies were different between mGFR_COR_ and mGFR the data were analysed using McNemar’s test.

Continuous variables that were normally distributed are reported as mean ± SD. Variables with non-normal distributions were reported as geometric mean ± SD and median [interquartile range (IQR)]. Categorical variables were reported as *n* (%).

## RESULTS

The study population characteristics are shown in Table 1. The mean age of the study population was 57.2 ± 12.2 years and 56% were women. The mean clearances, which were calculated using the warm and cold methods, are shown in Table 2.

**Table 1:** Study population characteristics.

Characteristics	Values
Participants, *N*	220
Age (years), mean ± SD	57.1 ± 12.2
Female, *n* (%)	124 (56)
Weight (kg), mean ± SD	79.3 ± 13.8
Length (cm), mean ± SD	173.2 ± 8.9
Body surface area (m^2^), mean ± SD	1.93 ± 0.2
BMI (kg/m^2^), mean ± SD	26.3 ± 3.5
Hypertension, *n* (%)	93 (42.3)
SBP (mmHg), mean ± SD	125.4 ± 12.7
DBP (mmHg), mean ± SD	73.9 ± 9.1
Medication use, *n* (%)	
Diuretics	20 (9.1)
RAAS inhibitors	41 (18.6)
Indication for GFR measurement, *n* (%)	
Kidney donor	191 (87%)
ADPKD research	29 (13%)
eGFR (ml/min/1.73 m^2^), mean ± SD	70.2 ± 23.0

eGFR: determined using creatinine; SBP: systolic blood pressure; DBP: diastolic blood pressure, RAAS: renin–angiotensin–aldosterone system.

**Table 2:** Overview of the calculated mGFR_COR_, mGFR and ERPF values.

Parameter	Values
mGFR_COR,warm_	86.4 ± 28.1
mGFR_warm_	83.7 ± 31.2
mGFR_COR,cold_	85.3 ± 29.4
mGFR_cold_	83.8 ± 32.1
ERPF_warm_	282.7 ± 90.3
ERPF_cold_	347.2 ± 122.9

All values are given as mean ± SD (ml/min).

The precision of mGFR_COR_ and mGFR was evaluated by the SD of the bias between mGFR_warm_ and mGFR_cold_. The precision was 8.04 ml/min (95% CI 6.94–9.02) for mGFR_COR_ and 4.40 ml/min (95% CI 3.92–4.82) for mGFR (*P* < .05). In addition, the intratest CV% was evaluated by comparing per-participant values obtained in the two urine collection periods. The intratest CV% of the corrected method was lower for both the warm (1.52 ± 3.53%) and cold (3.01 ± 3.15%) methods compared with the uncorrected method (5.50 ± 4.23% and 6.56 ± 3.44% for warm and cold, respectively), indicating the intended improvement in precision of the measurement method after correcting for incomplete bladder emptying. When comparing the warm and cold methods, both had a low intratest CV, with the warm method having a slightly lower intratest CV% for both mGFR_COR_ and mGFR as shown in Table 3.

**Table 3:** Intratest variability using results of the two urine collection periods assessed as the CV% for the corrected and uncorrected mGFR method and calculated for both the warm and cold methods (*N* = 220).

Intratest CV% warm method	Corrected	Uncorrected	*P*-value
Geometric mean (± SD)	1.52 ± 3.53	5.50 ± 4.23	<.0001
95% CI of geometric mean	1.28–1.79	4.54–6.66	
Median (IQR)	2.00 (1.01–3.20)	6.82 (2.94–13.02)	
Range	0.01–18.74	0.01–116.44	
Intratest CV% cold method	Corrected	Uncorrected	*P*-value
Geometric mean (± SD)	3.01 ± 3.15*	6.56 ± 3.44*	<.0001
95% CI of geometric mean	2.59–3.51	5.57–7.73	
Median (IQR)	3.71 (1.76–6.69)*	7.34 (3.10–15.33)*	
Range	0.01–29.82	0.11–117.31	

*P < .0001 when compared with the corresponding result for the warm method.

**Figure 1:**
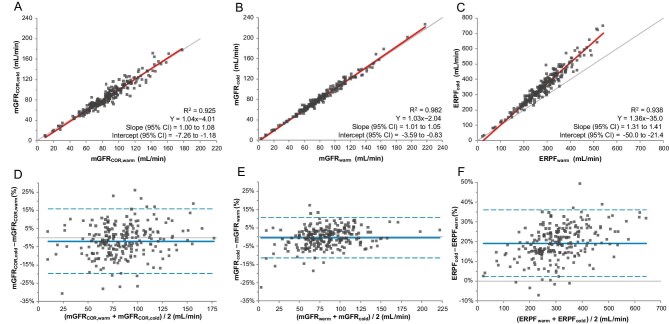
Passing–Bablok regression and Bland–Altman plots comparing the warm and cold methods. Passing–Bablok regression of the comparison between the warm and cold methods (*N* = 220) for **(A)** mGFR_COR_, **(B)** mGFR and **(C)** ERPF. The red line depicts the Passing–Bablok regression fit and the thin line shows the line of identity (*x* = *y*) for reference. Bland–Altman plot to determine agreement between the warm and cold methods for **(D)** mGFR_COR_, **(E)** mGFR and **(F)** ERPF. The solid blue line indicates the mean difference between the warm and cold measurements and the dashed blue lines show the 95% limits of agreement.

The warm method was compared with the cold method using Passing–Bablok regression analysis. The results are displayed in Fig. 1A–C. For mGFR_COR_, the results follow the line of identity, as the 95% CI of the slope was 0.99–1.07 and the 95% CI of the intercept ranged from −7.16 to −1.11 (Fig. 1A). For mGFR, the results also follow the line of identity, with the 95% CI of the slope between 1.01 and 1.05 and the 95% CI of the intercept between −3.60 and −0.82 (Fig. 1B). For ERPF however, the 95% CI of the slope was 1.30–1.41, indicating large proportional bias, and the 95% CI of the intercept was −48.7 to −20.3, indicating a large bias of the intercept (Fig. 1C).

The Bland–Altman plots provided in Fig. 1D and E show no indication for specific bias in the higher or lower range for mGFR_COR_ and mGFR. The mGFR_COR_ data were randomly scattered around zero, as the mean difference was −1.97% and the 95% CI was −3.18 to −0.77 (Fig. 1D). mGFR data are also randomly scattered around zero and showed a mean difference of −0.46% and a 95% CI of −1.21–0.29, reflecting agreement between the two methods (Fig. 1E). However, ERPF showed a clear bias, with a mean difference of 19.2% and a 95% CI of 18.0–20.3 and also an increased difference in ERPF values as the absolute ERPF values increased, when comparing the warm and cold methods (Fig. 1F). The Bland–Altman plots showing the absolute difference instead of the percentage difference are shown in Supplementary Fig. S9.

To assess accuracy, mGFR_COR_ values were compared between the warm and cold methods. The analysis showed a P_30_ of 100% (220/220 measurements with the cold method within 30% of the reference with the warm method) and a P_10_ of 76% (168/220 measurements within 10% of the reference), which both met the predefined acceptance criteria. For uncorrected mGFR, the accuracy was slightly higher, with a P_30_ of 100% (220/220 measurements within 30%) and a P_10_ of 94% (207/220 measurements within 10%). McNemar’s test showed that there was a significant difference between the P_10_ values of mGFR_COR_ and mGFR (*P* < .001).

These results for slope, intercept and accuracy were robust across various subgroups defined by sex, age, body mass index (BMI), indication and eGFR (Supplementary Tables S3 and S4).

Analysis of the ^131^I-hippuran formulation for impurities through HPLC separation and subsequent gamma counting showed three distinct peaks in the reconstructed chromatograms (Supplementary Fig. S3A and B). From left to right these are referred to as peaks 1, 2 and 3 in accordance with their order of elution. Based on their signal intensity and elution order (from polar to less polar, i.e. from lower to higher log P values), peaks 1, 2 and 3 were postulated to correspond to free ^131^I, ^131^I-hippuran and ^131^I-2-iodobenzoic acid, respectively. Subsequent Orbitrap LC-MS and ^1^H NMR analysis confirmed the presence of hippuran and 2-iodobenzoic acid in peaks 2 and 3, respectively (Supplementary Figs. S4–S8).

The radiochemical purity of ^131^I-hippuran was measured across different batches over time and the composition, on average, was 0.4% free ^131^I, 97.7% ^131^I-hippuran and 1.9% ^131^I-2-iodobenzoic acid (Supplementary Table S2).

## DISCUSSION

In this study it is shown that mGFR_COR_ and mGFR, determined using the cold method, both have high accuracy and precision when compared with the warm method, but that there are discrepancies when determining the ERPF. These results were robust across various subgroups. Moreover, we found radiochemical impurities in the ^131^I-hippuran formulation.

### Agreement between warm and cold methods for mGFR

The Bland–Altman analysis demonstrated statistical agreement between the cold and warm methods for mGFR with negligible mean bias seen between the two approaches. This indicates that, on average, the two methods yield highly similar results. For mGFR_COR_, the Bland–Altman analysis showed a small difference between the results obtained by the cold and warm methods. Additionally, Passing–Bablok regression analysis revealed slight proportional and constant biases for both mGFR_COR_ and mGFR. While these findings indicate statistically significant proportional and constant biases, these biases are minimal, average each other out in the range where most measurements are and therefore do not have clinical significance. Importantly, the minor differences in slope and intercept from the ideal values go in opposite directions, thus averaging each other out in the range of most measurements (i.e. the regression lines follow the line of identity in the range between 40 and 140 ml/min).

Importantly, the cold method achieved a P_30_ of 100% for mGFR_COR_ and mGFR, indicating that all measurements were within 30% of the reference values from the warm method. The P_10_ was 73% for mGFR_COR_ and 94% for mGFR. The P_10_ of mGFR_COR_ is higher compared with the P_10_ found when comparing the urinary clearance of inulin (gold standard) with the urinary clearance of iothalamate (73% versus 66%) [8]. These results confirm that the cold method is accurate for both mGFR_COR_ and mGFR assessment.

### Discordance in ERPF

In contrast to mGFR, ERPF results revealed a considerable discrepancy between the cold and warm methods, with the Passing–Bablok regression showing nearly all values above the line of identity, suggesting that the warm method results in substantially lower ERPF values compared with the cold method. Radiochemical impurities (free ^131^I and ^131^I-2-iodobenzoic acid) in the ^131^I-hippuran formulation can cause an underestimation in ERPF with the warm method [20–23]. The warm method, which measures total radioactivity, is biased by these impurities since hippuran is secreted through the organic anion transporters and is moved from the peritubular blood vessels into the tubular cells while both free iodide and 2-iodobenzoic acid are not [24]. Also, 2-iodobenzoic acid first accumulates in the liver and is then converted to hippuran before excretion [21, 22]. Consequently, these impurities are excreted more slowly than ^131^I-hippuran. A relatively high percentage of ^131^I or ^131^I-2-iodobenzoic acid in the formulation would contribute to higher radioactivity in serum samples relative to urine samples and infusion fluid, which, according to Equations (2) and (3), will lead to underestimation of the true ERPF [20, 23]. In this study the average radiochemical purity of the ^131^I-hippuran formulation was 97.7% with impurities accounting for 2.3% (free ^131^I: 0.4%; ^131^I-2-iodobenzoic acid: 1.9%) and this resulted in a 19% lower ERPF_warm_ compared with ERPF_cold_. Previous literature showed that 2% of free ^131^I causes a reduction in ERPF of 13% [20]. While routine practice involves purification of hippuran to achieve a radiochemical purity >99.5%, this purification is performed to reduce the amount of free ^131^I but is not aimed at reducing the amount of ^131^I-2-iodobenzoic acid, a known degradation product [25]. This finding demonstrates that although the amount of free ^131^I did not exceed 0.5%, the radiochemical purity of ^131^I-hippuran was lower than required due to the presence of ^131^I-2-iodobenzoic acid [20, 25]. Previous studies have reported variability in the composition of ^131^I-hippuran formulations and a reduction of purity over time [26–29]. Unfortunately, at this time, alternative formulations with higher radiochemical purity are unavailable. Importantly, the cold method is insensitive to the presence of these impurities as it specifically measured hippuran and therefore provides unbiased ERPF results. Summarizing, lower ERPF_warm_ results from radiochemical impurities, the slower urinary clearance of these impurities and the fact that the warm method cannot distinguish between the radioactivity of ^131^I-hippuran and that of ^131^I impurities.

### Correction for incomplete bladder emptying using the ERPF

Interestingly, the mGFR demonstrated even slightly higher accuracy than mGFR_COR_ in terms of P_10_ values (94% versus 76%), mean difference, a lower margin between the limits of agreement [(−11.5–10.5) versus (−19.7–15.8)], a higher *R*^2^ (0.982 versus 0.925) and a higher precision (4.40 versus 8.04 ml/min). The correction for incomplete bladder emptying, applied for the calculation of corrected mGFR, relies on hippuran clearance. Given the bias in ERPF measurements observed with the warm method, inaccuracies in hippuran quantification may propagate into the corrected mGFR, affecting its accuracy as well. When comparing the two clearance periods, the intratest variation was lower for mGFR_COR_ than for mGFR, both for the cold as well as for the warm method. This suggests that for both the warm and cold methods the precision of the measurement method is indeed improved by correcting for incomplete bladder emptying. The intratest CV% was slightly lower for the warm method compared with the cold method and this may be due to slightly better measurement precision resulting from the more complicated workflow for the cold method (extraction, chromatography and mass spectrometry) than for the warm method (gamma counting) [16].

One approach in analytical performance specifications states that the maximum allowable analytical variation should be based on the biological variation [30, 31]. Ideally the assay variation is small compared with the biological variation and thereby does not affect clinical decision-making [32]. The biological variation, based on the intertest CV% for two separate GFR measurements performed 3 months apart, of renal ^125^I-iothalamate clearance was determined to be 6.3% [33]. To minimize the impact of intratest variability on GFR outcome, the corrected method (for both the warm and cold methods) is preferable since after correction the intratest variability is (considerably) lower than the biological variation.

### Advantages of the cold method

The cold method offers several advantages over the warm method. By eliminating the use of radioactive tracers, the cold method reduces the risks associated with radiation exposure for both patients and healthcare personnel and could be utilized in a more diverse patient population (such as pregnant or breastfeeding patients, who are currently contraindicated) [34]. In addition, the non-radioactive method circumvents the logistical challenges of handling, shipping and disposing of radioactive materials and is likely to reduce costs in the long term.

### Limitations and future perspectives

This study has some limitations. The potential biases inherent in the warm method, particularly those related to radiochemical impurities, underscore the importance of validating the cold method against other exogenous filtration markers. Second, our study was performed as a single-centre cohort study in kidney donors and patients with ADPKD. External validation in diverse patient populations is needed to corroborate that cold GFR measurement is applicable in a broader patient population for which assessment of kidney function by measuring GFR is indicated [3].

## CONCLUSION

Our study demonstrates that the recently developed cold method for mGFR measurement provides equivalent results compared with the warm method. The cold method offers significant advantages in terms of safety, accessibility and practicality and also avoids inaccuracies due to radiochemical impurities in ERPF measurements.

## Supplementary Material

sfaf293_Supplemental_File

## Data Availability

Data will be made available upon request to the corresponding author.
