# Supplementary material for: Comparison of radioactive and non-radioactive iothalamate and hippuran to assess kidney function
Source: Clin Kidney J. 2025 Sep 23;18(10):sfaf293. doi: 10.1093/ckj/sfaf293 (PMC12527239; doi:10.1093/ckj/sfaf293)
Supplement: sfaf293_Supplemental_File [file sfaf293_supplemental_file.docx]

Contents

[Supplementary data 2](#_Toc206094594)

[1. Quantification of iothalamate and hippuran in infusion solution 2](#_Toc206094595)

[1.1. Method 2](#_Toc206094596)

[1.1.1. Standard solutions, calibration and validation samples 2](#_Toc206094597)

[1.1.2. Sample preparation 2](#_Toc206094598)

[1.1.3. Validation 2](#_Toc206094599)

[1.2. Validation results 3](#_Toc206094600)

[1.2.1. Accuracy and precision 3](#_Toc206094601)

[1.2.2. Carry-over 3](#_Toc206094602)

[1.2.3. Stability and reinjection reproducibility 3](#_Toc206094603)

[2. Long-term storage stability in serum and urine 3](#_Toc206094604)

[3. Radiochemical purity 6](#_Toc206094605)

[HPLC 6](#_Toc206094606)

[Orbitrap LC-MS 9](#_Toc206094607)

[^1^H NMR 13](#_Toc206094608)

[4. Comparison of warm and cold method in absolute values (mL/min) 16](#_Toc206094609)

[5. Subgroup analysis 18](#_Toc206094610)

[6. References 20](#_Toc206094611)

# Supplementary data

## Quantification of iothalamate and hippuran in infusion solution

The method used for the detection of iothalamate and hippuran in infusion solution was identical in terms of liquid chromatography-tandem mass spectrometry (LC-MS/MS) settings as reported previously for serum and urine (1), but was slightly different with regard to sample pretreatment and also used other analyte concentrations, as described below:

### Method

The infusion solvent was composed of 0.9% saline (9 grams of sodium chloride (Sigma Aldrich, St. Louis, MO, USA) dissolved in 1 liter of ultrapure water, which was subsequently diluted with tap water in a ratio of 1:50 or 1:100 (v/v) to mimic the initial handling of the infusion solution samples in the clinic.

### Standard solutions, calibration and validation samples

The calibration samples were prepared in a mixture of 0.9% saline and with tap water (1:100, v/v), the calibration levels were at 5.00, 10.0, 25.0, 50.0, 100, and 1000 ng/mL for both analytes.

Samples used for validation were unspiked (blank) infusion solution (0.9% saline and tap water, 1:100 v/v), and quality control samples spiked at four concentrations: lower limit of quantification (LLOQ), LOW, MEDIUM (MED), HIGH, which were 5.00, 15.0, 300 and 750 ng/mL, respectively. In addition, the LOW and HIGH QC samples were also prepared in a mixture of 0.9% saline and tap water in a ratio of 1:50 (v/v) and in an undiluted 0.9% saline solution.

The internal standard working solution was prepared in a methanol/water mixture (10:90, v/v) at 2.625 ng/mL.

### Sample preparation

The analyte concentrations in infusion solution are relatively high and, for this reason, these samples are already diluted (1:100 or 1:50, v/v) with tap water in the clinic. To bring the concentration within the linear dynamic range of the mass spectrometer a further 20-fold dilution with internal standard working solution was performed.

The sample preparation consisted of a single dilution step. Aliquots of 20 µL of well-homogenized infusion solution were pipetted into a 1.0-mL 96 deep well plate and 400 µL of internal standard working solution was added. After vortex-mixing for 1 minute at 1300 rpm the plate was capped and the extracts were stored at 4 °C until injection.

### Validation

Method validation included assessment of precision and accuracy, carryover, stability, and reinjection reproducibility as previously described (1). In short:
Precision and accuracy were evaluated at the four concentrations (LLOQ, LOW, MED, HIGH) using QC samples analyzed in six-fold across three runs. Within-run, between-run, and total CVs were calculated by analysis of variance. Carryover was tested by analyzing three blanks following the highest calibration standard or the HIGH QC. Stability at LOW and HIGH levels was assessed in triplicate under various conditions: five freeze/thaw cycles, 24 h at ambient temperature, long-term storage at -20 °C for 503 days, and autosampler storage at 4 °C for 8 days. Reinjection reproducibility was determined by reanalyzing stored calibration and QC samples after 8 days at 4 °C.

### Validation results

### Accuracy and precision

Upon analysis of the validation samples in six-fold in three analytical runs, values for CV and bias not exceeding 4% were obtained for both iothalamate and hippuran as shown in supplemental table 1.

Supplemental table 1 The resulting precision (CV) and accuracy (bias) of the methods for iothalamate and hippuran

| Matrix | Concentration (ng/mL) | Within-Run CV (%) | Between-Run CV (%) | Overall CV (%) | Overall bias (%) |
| --- | --- | --- | --- | --- | --- |
| Iothalamate | | | | | |
| Infusion solution | LLOQ (5.00) | 2.2 | 2.1 | 3.1 | 1.5 |
|  | LOW QC (15.0) | 2.7 | 2.5 | 3.7 | 0.8 |
|  | MED QC (300) | 3.0 | 0.0 | 3.0 | -2.3 |
|  | HIGH QC (750) | 2.3 | 1.9 | 3.0 | 3.4 |
| Hippuran | | | | | |
| Infusion solution | LLOQ (5.00) | 2.0 | 2.8 | 3.5 | 2.1 |
|  | LOW QC (15.0) | 1.2 | 2.4 | 2.7 | -1.3 |
|  | MED QC (300) | 1.4 | 0.0 | 1.4 | -0.5 |
|  | HIGH QC (750) | 1.2 | 3.0 | 3.2 | 0.1 |

For all concentrations n = 18

### Carry-over

No unacceptable carry-over was found for any of the blank samples: analyte signals were always <20% of the corresponding response at the LLOQ level.

### Stability and reinjection reproducibility

The analytes were found stable in infusion solution (0.9% saline and tap water, 1:100 v/v), at room temperature for up to 24 hours and through five complete freeze-thaw cycles between -20 °C and room temperature. Long-term stability at -20 °C was determined for 503 days. Autosampler stability at 4 °C was determined for up to 8 days. Acceptable reinjection reproducibility was established after storage of sample extracts for 8 days.

## Long-term storage stability in serum and urine

Previously, long-term stability of iothalamate and hippuran in serum and urine at -20 °C was determined for a period of up to 200 days (1). In the current investigation, this period was extended to 540 days and 519 days, for serum and urine respectively.

Also, the results for mGFR and ERPF, obtained by the “cold” (LC-MS/MS) method, were compared with the corresponding results obtained by the “warm” reference method for two situations: cold analysis of patient samples that had been stored at -20 °C for 500 days and cold analysis of samples that had been stored at -20 °C for no longer than 3 weeks, in both cases against the results of warm analysis immediately after sample collection (supplemental figures 1 and 2). The mGFR and ERPF results for samples analyzed by LC-MS/MS after 500 days of storage showed a similar trend as the samples analyzed after 3 weeks of storage, which gives additional evidence that long-term storage of serum and urine does not affect the measurement outcome.


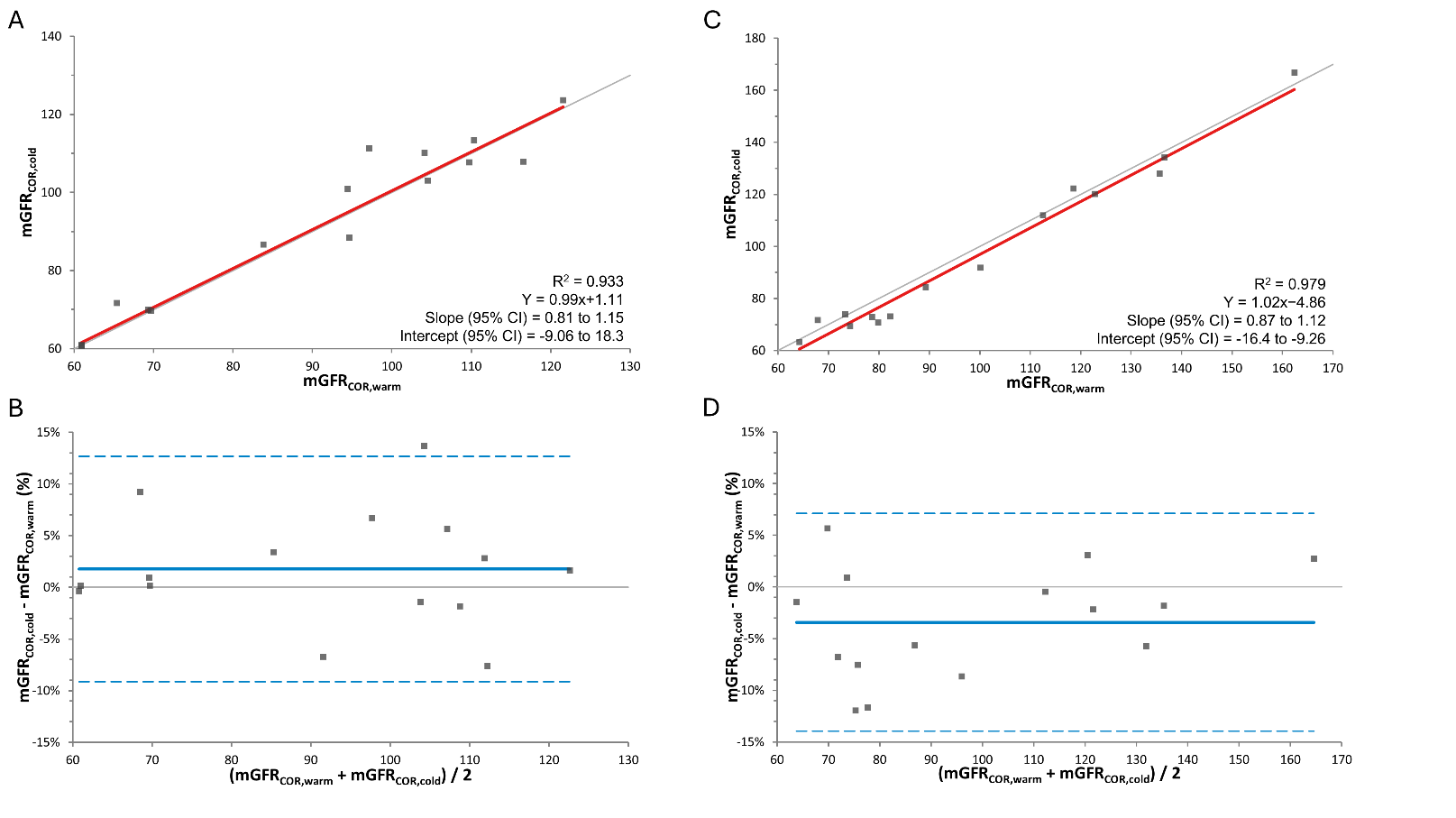


Supplemental figure 1 Passing-Bablok regression and (A) Bland-Altman plot (B) of the mGFR comparison using the warm and cold method of samples stored for 2-3 weeks (n=15) and the Passing-Bablok regression and (C) Bland-Altman plot (D) of the mGFR comparison of samples stored for 500 days. The red line depicts the Passing-Bablok regression fit and the thin line shows the line of unity (x=y) for reference. For the Bland-Altman plots the solid blue line indicates the mean difference between the warm and cold measurements and the dashed blue lines show the 95% Limits of Agreement.


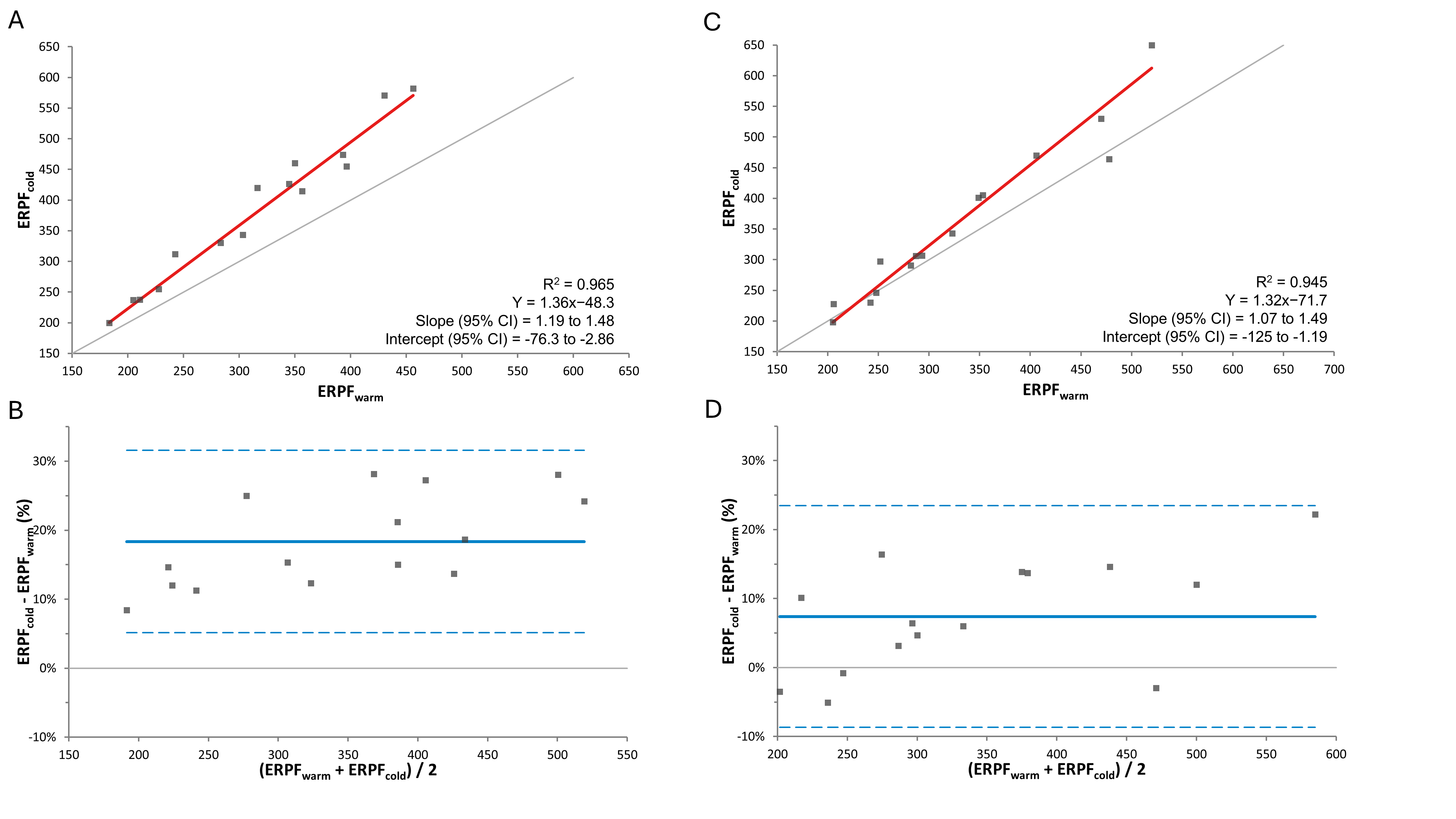


Supplemental figure 2 Passing-Bablok regression and (A) Bland-Altman plot (B) of the ERPF comparison using the warm and cold method of samples stored for 2-3 weeks (n=15) and the Passing-Bablok regression and (C) Bland-Altman plot (D) of the ERPF comparison of samples stored for 500 days. The red line depicts the Passing-Bablok regression fit and the thin line shows the line of unity (x=y) for reference. For the Bland-Altman plots the solid blue line indicates the mean difference between the warm and cold measurements and the dashed blue lines show the 95% Limits of Agreement.

## Radiochemical purity

The radiochemical purity was determined using high-performance liquid chromatography (HPLC), Orbitrap LC-MS and ^1^H nuclear magnetic resonance (NMR).

### HPLC

The possible presence of impurities in the ^131^I-hippuran formulation was assessed using HPLC with off-line radioactivity detection. It was performed using a Phenomenex (Torrance, CA, USA) Luna NH₂ column (250 × 10 mm internal diameter, 5 µm particle size). The mobile phase consisted of a mixture of 0.2% phosphoric acid in water and acetonitrile (90:10, v/v) and isocratic elution was performed at a flow rate of 5 mL/min. A 2-mL sample of the ^131^I-hippuran formulation was injected and fractions of the column effluent were collected every 30 seconds using a Foxy R1 Teledyne ISCO fraction collector. Detection of radioactivity in these fractions was performed using the 2470 WIZARD^2^ gamma counter (Waltham, MA, USA) and a chromatogram was reconstructed by plotting the ^131^I radioactivity levels (in counts per minute, CPM) versus collection time (supplemental figures 3A and 3B). The radiochemical purity of the injected ^131^I-hippuran formulation was quantified by determining the radioactivity signals corresponding to three chromatographic peaks that were observed (peaks 1, 2 and 3) and calculating the percentage of the radioactivity of each peak in comparison to the total radioactivity of the three peaks combined.


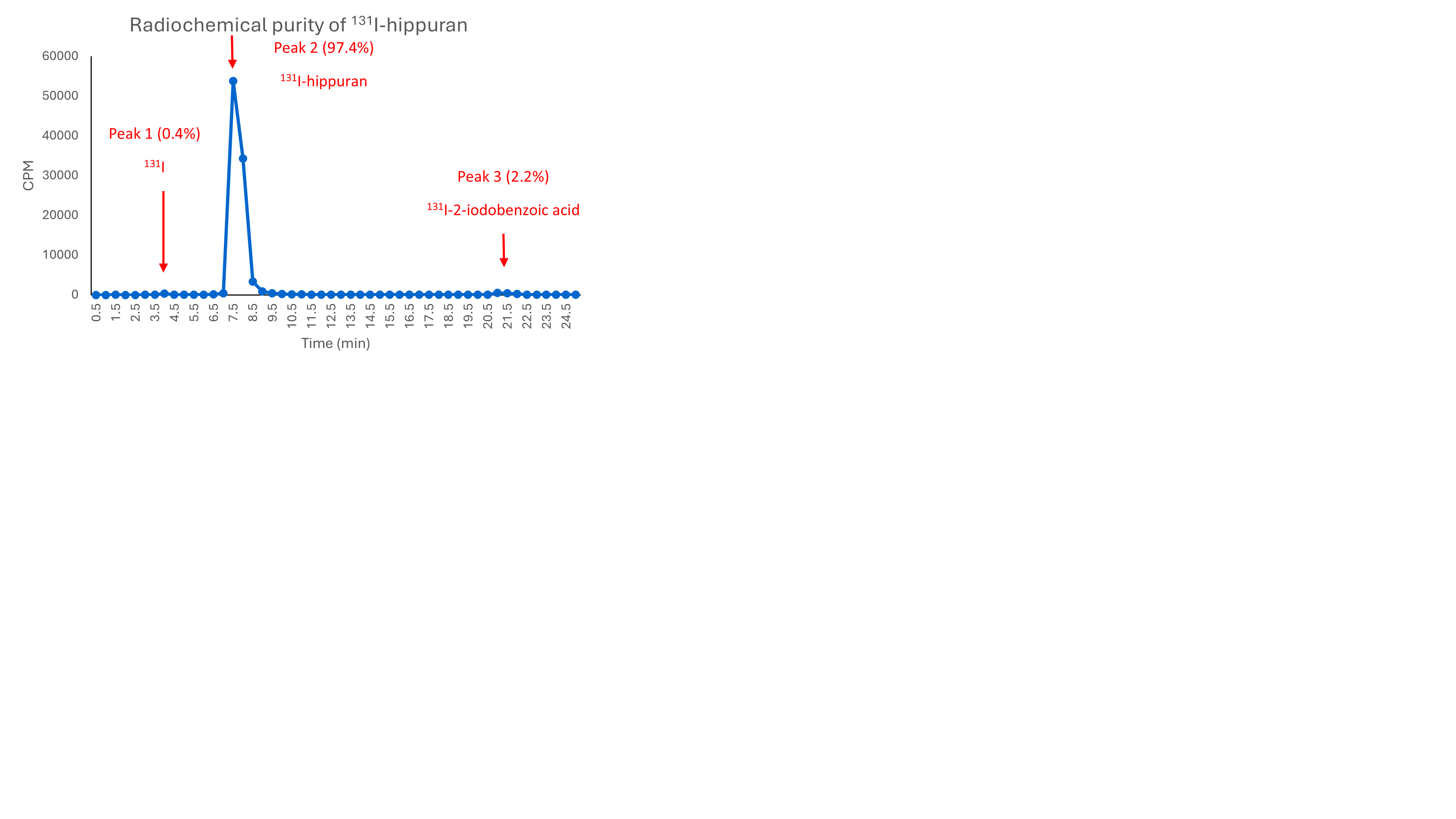


Supplemental figure 3A Example chromatogram after injection of ^131^I-hippuran formulation and separation using HPLC. The signal that is measured is the number of counts per minute (CPM) of ^131^I radioactivity


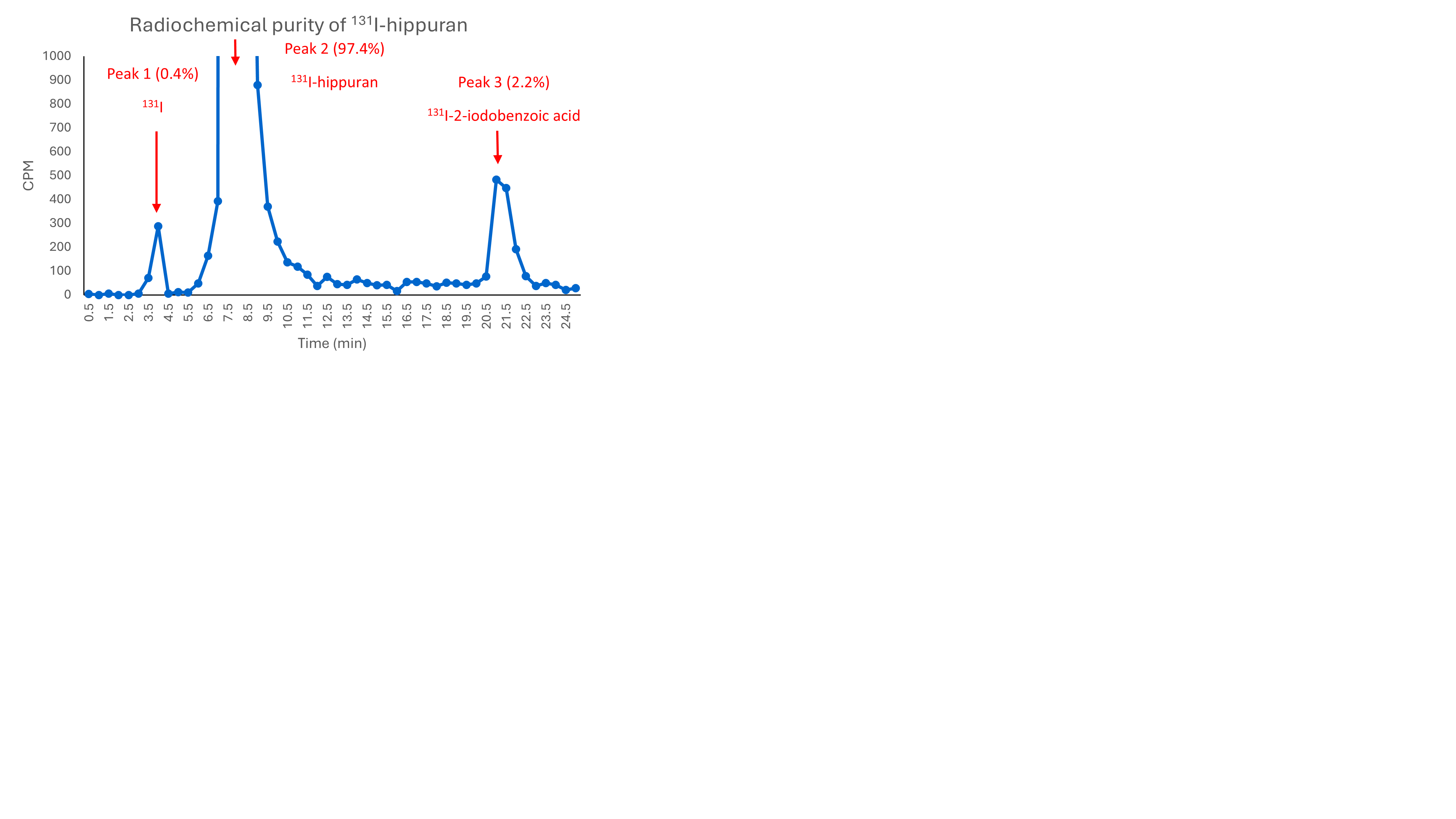


Supplemental figure 3B The chromatogram of supplemental figure 3A zoomed in, to better visualize peaks 1 and 3.

Based on their elution order and relative intensities, peaks 1, 2 and 3 were hypothesized to be free ^131^I, ^131^I-hippuran and the possible in vitro degradation product ^131^I-2-iodobenzoic acid, respectively. The radiochemical composition was measured in different batches of ^131^I-hippuran over time. Results are shown in supplemental table 2. On average the proportion of the compounds was: 0.4% for free ^131^I, 97.7% for ^131^I-hippuran, and 1.9% for ^131^I-2-iodobenzoic acid.

Supplemental table 2 The radiochemical purity measured in different batches of the ^131^I-hippuran formulation over time.

| ^131^I-hippuran batch (number of purifications) | Time between purification and analysis (days) | ^131^I (%) | ^131^I-Hippuran (%) | ^131^I-2-iodobenzoic acid (%) |
| --- | --- | --- | --- | --- |
|  |  |  |  |  |
| Batch A (1) | 4 | 0.39 | 96.35 | 3.26 |
| Batch A (2) | 0 | 0.38 | 98.27 | 1.34 |
| Batch B (0) | 0 | 0.35 | 98.01 | 1.64 |
| Batch B (1) | 0 | 0.19 | 98.42 | 1.40 |
| Batch B (1) | 11 | 0.40 | 97.90 | 1.71 |
| Batch C (0) | 0 | 0.40 | 98.60 | 0.96 |
| Batch C (1) | 0 | 0.40 | 97.90 | 1.70 |
| Batch D (0) | 0 | 0.48 | 96.60 | 2.92 |
| Batch D (1) | 3 | 0.35 | 97.50 | 2.15 |
| Batch D (1) | 7 | 0.31 | 97.54 | 2.15 |
| Batch E (0) | 0 | 0.69 | 97.13 | 2.18 |
| Batch E (1) | 0 | 0.33 | 98.56 | 1.11 |
| Batch E (1) | 4 | 0.30 | 98.76 | 0.95 |
| Batch E (1) | 11 | 0.37 | 98.60 | 1.03 |
| Batch F (0) | 0 | 0.65 | 95.40 | 3.95 |
| Batch F (1) | 0 | 0.27 | 98.01 | 1.72 |

### Orbitrap LC-MS

The structural identification of the different components from the formulation was performed using a Vanquish UHPLC coupled to a Q Exactive Orbitrap mass spectrometer (Thermo Fisher Scientific , San Jose, CA, USA). Chromatographic separation was performed on a Waters (Milford, MA, USA) HSS T3 column (100 mm × 2.1 mm internal diameter, 1.8 µm particle size). Mobile phase A consisted of 0.01% acetic acid in water, mobile phase B was 100% acetonitrile and a gradient was run with a flow of 0.500 mL/min, starting at 100% solvent A and 0% solvent B, for one minute, then changing linearly to 0% A and 100% B at 10.0 min; this composition was maintained until 11.5 min, after which it linearly changed back to 0% B at 12.5 min. The samples that were analyzed corresponded to peaks 1, 2 and 3 from the HPLC separation, and were the fractions collected from 3.5 to 4.0 min, 7.5 to 8.0 min and 20.5 to 21.0 min after injection, respectively (as indicated in supplemental figure 3A-B). The injection volume was 5 µL for all samples. The MS settings were as follows: CID at 0.0 eV, resolution at 35000, maximum IT at 100 ms and the scan range from 120 to 400 m/z. The dd-MS² settings were: resolution at 17500, maximum IT at 64 ms and the normalized collision energy applied was 35 V. The ion source settings (in negative mode) were: spray voltage 3100 V, sheath gas 50 arbitrary units, auxiliary gas 15 arbitrary units, ion transfer tube temperature 380 °C and vaporizer temperature 350 °C.

Two pure reference standards were used to support the identification: hippuran and 2-iodobenzoic acid. Supplemental figure 4 shows the full scan spectra recorded for 1-mg/L solutions of these standards in negative ionization mode. For both analytes, the [M-H]^-^ ions (m/z 303.948 for hippuran and m/z 246.926 for 2-iodobenzoic acid) were most abundant, which closely corresponds to the predicted monoisotopic values of 303.958 and 246.926, respectively. These m/z values were used for the analysis of the samples corresponding to peaks 1, 2 and 3. Mass extraction windows of ±0.002 mass units around the mean were used to construct extracted ion chromatograms (EIC).


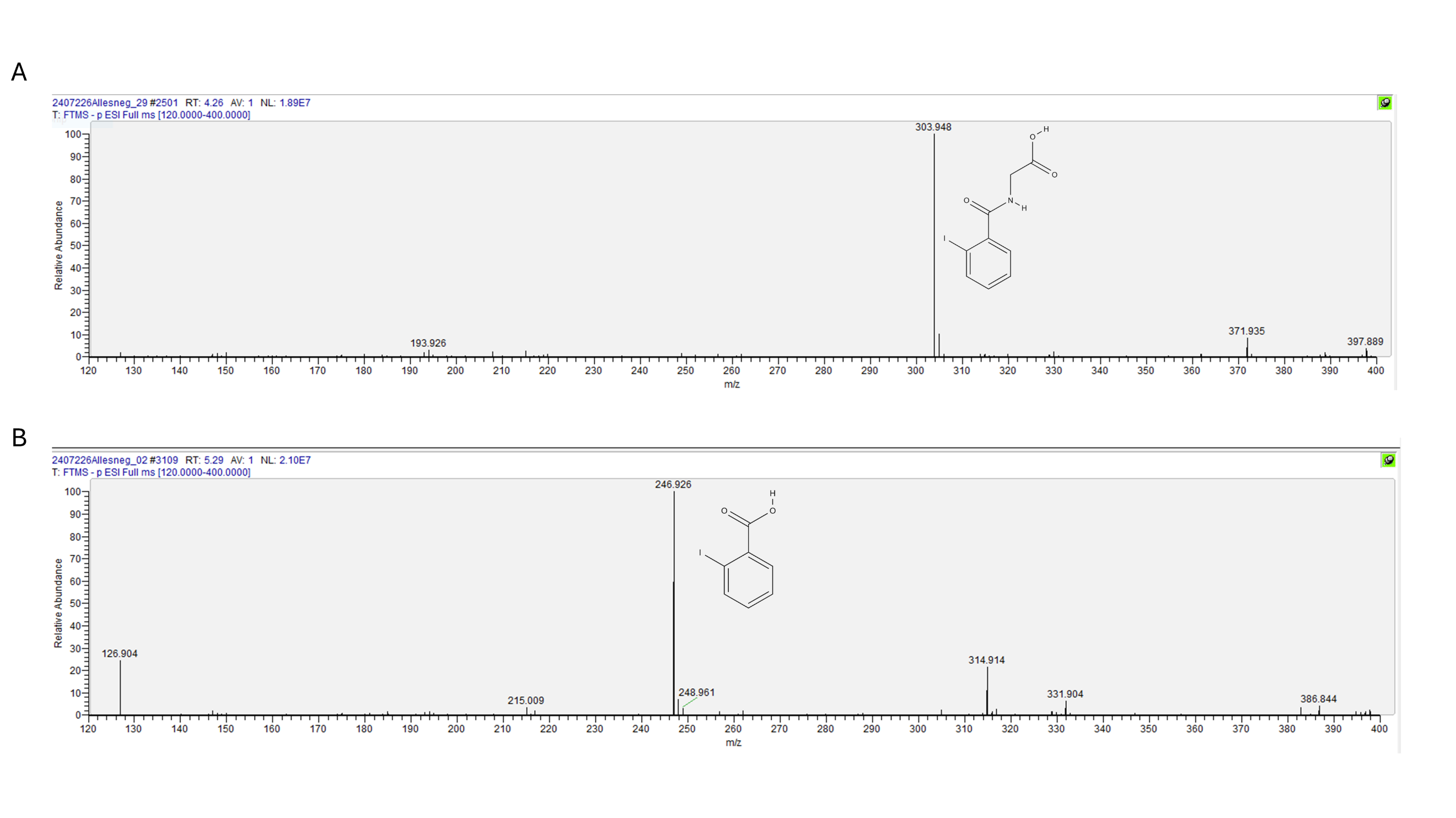


Supplemental figure 4 Full scan spectra showing the most abundant precursor ion for a standard solution of hippuran at m/z 303.948. (A) and for a standard solution of 2-iodobenzoic acid at m/z 246.926 (B).


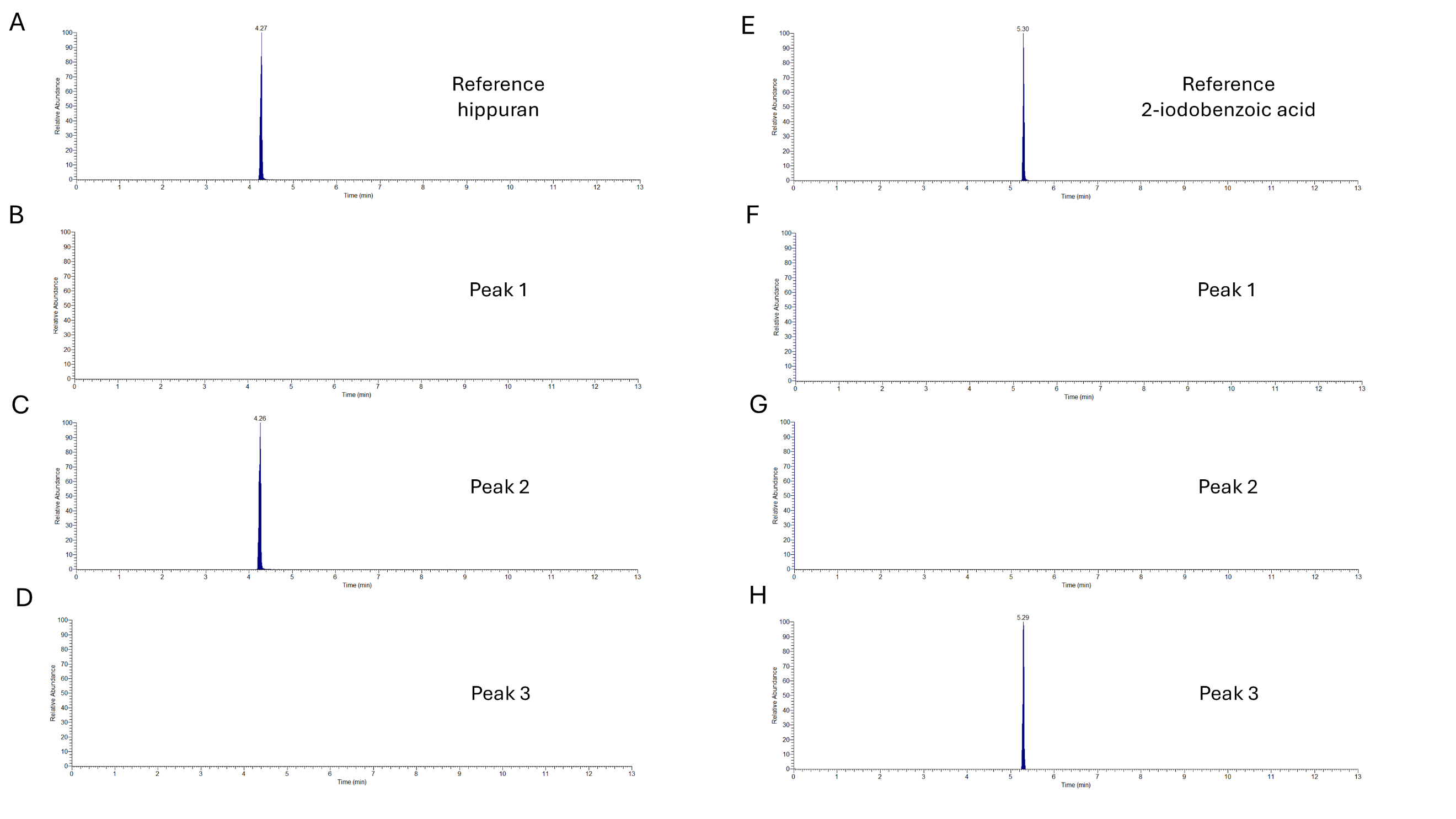


Supplemental figure 5: Orbi-trap-MS extracted-ion chromatograms (EIC) of (left-hand-side) hippuran in from top to bottom: the 1-mg/L standard solution (A), peak 1 (B), peak 2 (C) and peak 3 (D) and (right-hand side) 2-iodobenzoic acid in from top to bottom: the 1-mg/L standard solution (E), peak 1 (F), peak 2 (G) and peak 3 (H).

As shown in supplemental figure 5, EICs with the mass windows m/z 303.946-303.950 and m/z 246.924-246.928 were recorded for hippuran and 2-iodobenzoic acid, respectively. These chromatograms show that the sample corresponding to peak 2 isolated by HPLC from the formulation contains a compound that has both the same retention time (4.27 min) and the same exact mass as hippuran (supplemental figure 5C), while the sample corresponding to peak 3 contains a compound that has the same retention time (5.30 min) and the same exact mass as 2-iodobenzoic acid (supplemental figure 5H). This convincingly confirms that peak 2 contains hippuran and peak 3 contains the impurity 2-iodobenzoic acid.

### ^1^H NMR

NMR spectra were recorded using a JNM-ECZL400S Spectrometer (JEOL, Tokyo, Japan) (^1^H NMR at 400 MHz and 9.4 T) at 293 K. Chemical shifts (δ) were reported in ppm. All chemical shifts were analyzed in correspondence to the solvent peak (DMSO-d_6_). Five samples were analyzed: peak 1, peak 2, peak 3, dissolved (pure) hippuran and dissolved (pure) 2-iodobenzoic acid.

#### Peak 3


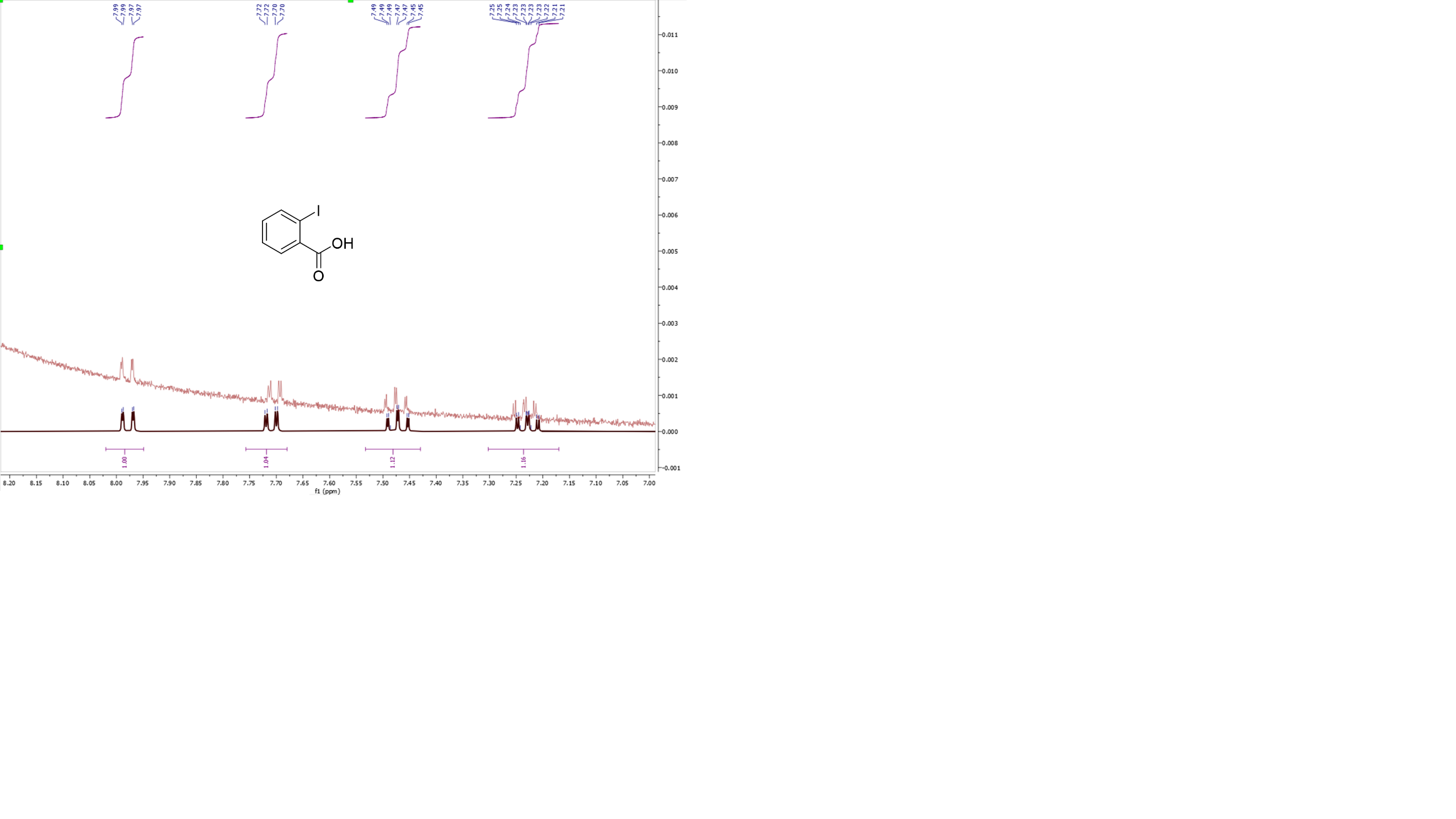


Supplemental figure 6: ^1^H NMR spectrum of 2-iodobenzoic acid (bottom) overlapped with the spectrum generated for peak 3

The identity of the impurity postulated to be 2-iodobenzoic acid was further confirmed through NMR analysis of the sample corresponding to peak 3 and its comparison to the analysis results of pure 2-iodobenzoic acid (supplemental figure 6). The NMR data of both samples are very similar, with the aromatic region (7.2-8.0 ppm) exhibiting four distinct signals corresponding to the four aromatic protons of the compound's benzene ring, which is in agreement with the expected substitution pattern of 2-iodobenzoic acid. The absence of non-aromatic signals (e.g., CH₂ or NH) strongly suggests that the spectrum does not belong to hippuran: ^1^H NMR (400 MHz, DMSO-*D*_6_) δ 7.94 (dd, *J* = 7.9, 1.2 Hz, 1H), 7.67 (dd, *J* = 7.7, 1.8 Hz, 1H), 7.44 (td, *J* = 7.5, 1.2 Hz, 1H), 7.22 – 7.17 (m, 1H) ppm. The analysis of chemical shifts, splitting patterns, and integration further supports the conclusion that the compound present in the sample corresponding to peak 3 is in fact 2-iodobenzoic acid (2).

#### Peak 2


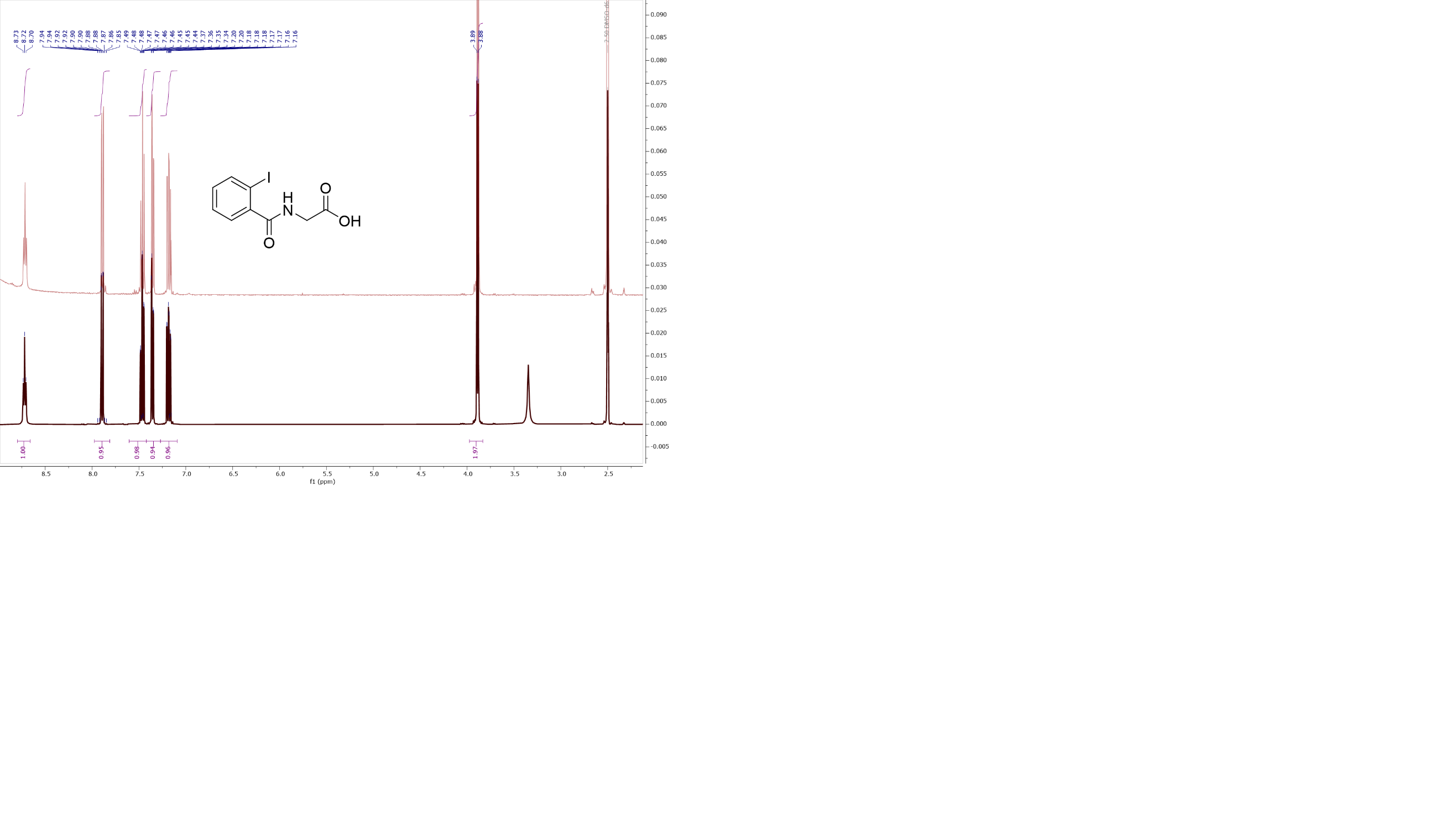
Supplemental figure 7: ^1^H NMR spectrum of hippuran (bottom) overlapped with the spectrum generated for peak 2

The NMR results for peak 2 (postulated to be hippuran) are shown in supplemental figure 7. This compound was indeed identified as hippuran since its spectrum is identical to the one obtained for pure hippuran and contains the predicted signals for additional non-aromatic protons from the hippuric acid side chain: ^1^H NMR (400 MHz, DMSO-*D*_6_) δ 8.71 (t, *J* = 6.0 Hz, 1H), 7.89 (dd, *J* = 7.9, 1.1 Hz, 1H), 7.46 (td, *J* = 7.5, 1.1 Hz, 1H), 7.35 (dd, *J* = 7.6, 1.7 Hz, 1H), 7.21 – 7.15 (m, 1H), 3.88 (d, *J* = 6.0 Hz, 2H) ppm (3). This is confirmed by the -CH₂ group (glycine moiety) indicated by a singlet or weakly split signal around 4.0-4.5 ppm (adjacent to the nitrogen atom) and the NH proton indicated by a broad singlet or exchangeable peak between 7.5-9.0 ppm.

#### Peak 1


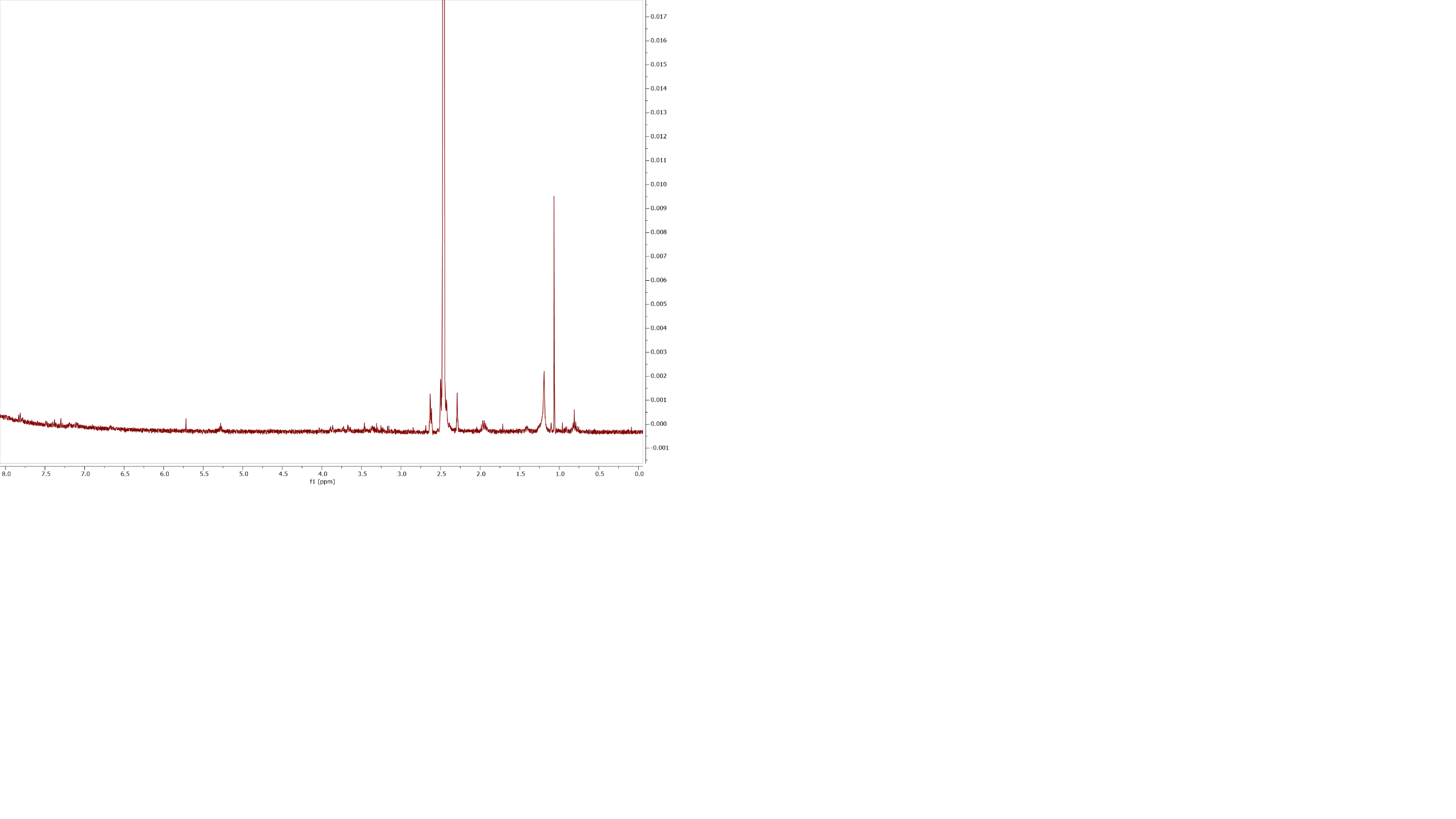
Supplemental figure 8: ^1^H NMR spectrum for peak 1.

The NMR spectrum obtained for the sample corresponding to peak 1 (postulated to be free iodine) is shown in supplemental figure 8 and does not show any distinct signal apart from the one generated by DMSO. This could very well correspond to a solution containing iodine, since no hydrogen atoms are present that could be detected.

##
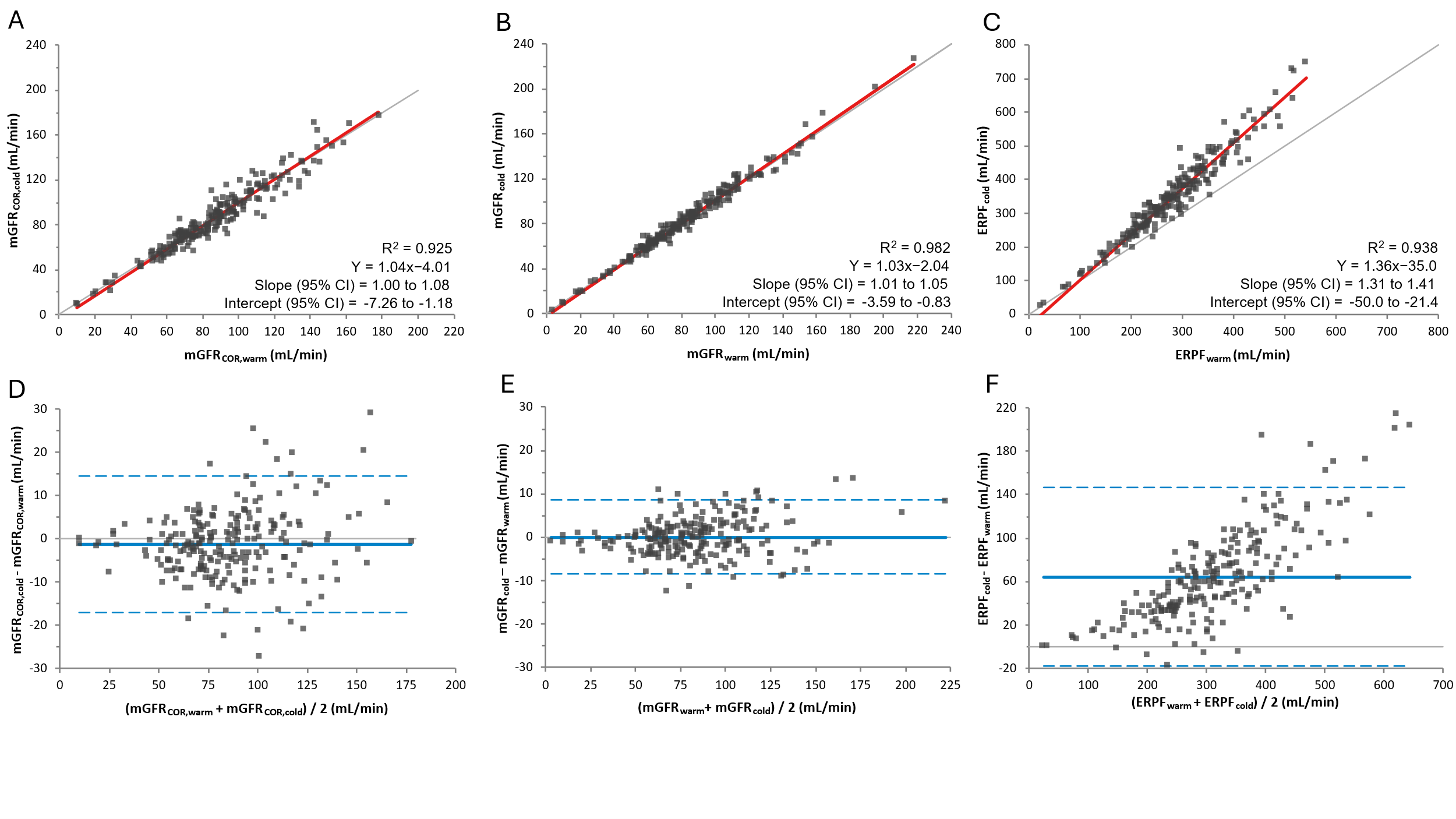
Comparison of warm and cold method in absolute values (mL/min)

Supplemental figure 9 Passing-Bablok regression and Bland-Altman plots comparing the warm and cold method

Passing-Bablok regression of the comparison between the warm and cold method (N = 220) for mGFR_COR_ (A), mGFR (B) and ERPF (C). The thick line depicts the Passing-Bablok regression fit and the thin line shows x=y (line of unity for reference). Bland-Altman plot to determine agreement between the warm and cold method for mGFR_COR_ (D), mGFR (E) and ERPF (F). The thick solid line indicates the mean difference between the warm and cold measurements (in mL/min) and the dashed lines show the 95% Limits of Agreement.

In supplemental figure 9 the Passing-Bablok regression and Bland-Altman plots are shown for the comparison of the warm and cold method. In this figure the Bland-Altman plots show the absolute difference on the y-axis instead of the percentage difference. The Bland-Altman plots provided in supplemental figure 9 (D-E) showed no indication for specific bias in the higher or lower range for mGFR_COR_ and mGFR. The mGFR_COR_ data were randomly scattered around 0, the mean difference was -1.15 mL/min and the 95% CI was between -2.22 and -0.08 (supplemental figure 9D). mGFR data are also randomly scattered around 0 and showed a mean difference of 0.10 mL/min and a 95% CI between -0.48 and 0.69, reflecting agreement between the two methods (supplemental figure 9E). However, ERPF showed a clear bias with a mean difference of 64.1 mL/min and a 95% CI between 58.9 and 70.1 and also an increased difference in ERPF values as the absolute ERPF values increased, when comparing the warm and cold method (supplemental figure 9F).

## Subgroup analysis

For the subgroup analysis age, BMI and eGFR were each separated into two groups which were equally split at the median (as shown in supplemental table 3 and 4). An independent (unpaired) t-test was performed to compare the mean difference.

Supplemental table 3 Analysis of the mGFR results, when comparing the warm and cold method, in terms of mean difference across various subgroups defined by sex, age, BMI, indication, and eGFR

| Subgroup | Group 1 | Group 2 | Mean Difference (%) | P-value |
| --- | --- | --- | --- | --- |
| Sex | Female (N=124) | Male (N=96) |  |  |
|  | -0.51 | -0.40 | -0.104 | 0.894 |
| Age | ≥57.9 (N=110) | <57.9 (N=110) |  |  |
|  | -0.33 | -0.59 | 0.268 | 0.724 |
| BMI | ≥25.9 (N=110) | <25.9 (N=110) |  |  |
|  | -0.83 | -0.09 | -0.743 | 0.327 |
| Indication | ADPKD (N=29) | Kidney donor (N=191) |  |  |
|  | -2.40 | -0.17 | -2.233 | 0.152 |
| eGFR | ≥75.9 (N=109) | <75.9 (N=109) |  |  |
|  | -0.22 | -0.74 | 0.523 | 0.493 |

Supplemental table 4 Analysis of the mGFR results, when comparing the warm and cold method, in terms of slope, intercept and accuracy across various subgroups defined by sex, age, BMI, indication, and eGFR

| Comparison | Subgroups | N | P_10_ (%) | P_30_ (%) | Intercept |  | Slope |  |
| --- | --- | --- | --- | --- | --- | --- | --- | --- |
| Sex | Female | 124 | 95.2 | 100.0 | -1.91 |  | 1.027 |  |
|  | Male | 96 | 92.7 | 100.0 | -1.51 |  | 1.022 |  |
| Age | ≥57.9 | 110 | 92.7 | 100.0 | -2.24 |  | 1.036 |  |
|  | <57.9 | 110 | 95.5 | 100.0 | -2.44 |  | 1.034 |  |
| BMI | ≥25.9 | 110 | 97.3 | 100.0 | -2.39 |  | 1.017 |  |
|  | <25.9 | 110 | 90.9 | 100.0 | -2.59 |  | 1.045 |  |
| Indication | ADPKD | 29 | 86.2 | 100.0 | -1.33 |  | 1.024 |  |
|  | Kidney donors | 191 | 95.3 | 100.0 | -2.63 |  | 1.036 |  |
| eGFR | ≥75.9 | 109 | 98.2 | 100.0 | -5.46 |  | 1.062 |  |
|  | <75.9 | 109 | 89.9 | 100.0 | -2.52 |  | 1.043 |  |

## References

1. Mohamed AAA, Walland P, Stevens J et al. A validated LC-MS/MS method for the simultaneous quantification of iothalamate and hippuran in serum and urine for non-radioactive kidney function assessment. Journal of Chromatography B *[Internet]* 2024; 1247: 124329. Available from: https://linkinghub.elsevier.com/retrieve/pii/S1570023224003386

2. Patel SS, Patel DB, Poddar AK, Patel DA, Kotadiya DD, Patel HD. Copper Phthalocyanine Tetrasulfonic Acid, a Recyclable Solid Acid Catalyst for the Synthesis of Aryl Iodides and Azides from Aryl Amines via Diazotization. Organic Preparations and Procedures International *[Internet]* 2024; [cited 2025 May 14] Available from: https://www.tandfonline.com/doi/abs/10.1080/00304948.2024.2355433

3. Fabry DC, Stodulski M, Hoerner S, Gulder T. Metal-Free Synthesis of 3,3-Disubstituted Oxoindoles by Iodine(III)-Catalyzed Bromocarbocyclizations. Chemistry – A European Journal *[Internet]* 2012; [cited 2025 May 14] 18: 10834–10838. Available from: /doi/pdf/10.1002/chem.201201232
